# Mobile Texting and Lay Health Supporters to Improve Schizophrenia Care in a Resource-Poor Community in Rural China (LEAN Trial): Randomized Controlled Trial Extended Implementation

**DOI:** 10.2196/22631

**Published:** 2020-12-01

**Authors:** Yiyuan Cai, Wenjie Gong, Hua He, James P Hughes, Jane Simoni, Shuiyuan Xiao, Stephen Gloyd, Meijuan Lin, Xinlei Deng, Zichao Liang, Wenjun He, Bofeng Dai, Jing Liao, Yuantao Hao, Dong (Roman) Xu

**Affiliations:** 1 School of Public Health Sun Yat-sen University Guangzhou China; 2 School of Public Health Guizhou Medical University Guiyang China; 3 Xiangya School of Public Health Central South University Changsha China; 4 School of Public Health and Tropical Medicine Tulane University New Orleans, LA United States; 5 Department of Biostatistics University of Washington Seattle, WA United States; 6 Department of Psychology University of Washington Seattle, WA United States; 7 Department of Global Health University of Washington Seattle, WA United States; 8 Acacia Lab for Health Systems Strengthening and Department of Health Management School of Health Management Southern Medical University Guangzhou China

**Keywords:** medication adherence, mobile texting, lay health worker, resource-poor community, primary health care, quality of care, mHealth, schizophrenia

## Abstract

**Background:**

Schizophrenia is a severe and disabling condition that presents a dire health equity challenge. Our initial 6-month trial (previously reported) using mobile texting and lay health supporters, called LEAN, significantly improved medication adherence from 0.48 to 0.61 (adjusted mean 0.11, 95% CI 0.03 to 0.20, *P*=.007) for adults with schizophrenia living in a resource-poor village in rural China.

**Objective:**

We explored the effectiveness of our texting program in improving participants’ medication adherence, functioning, and symptoms in an extended implementation of the intervention after its initial phase.

**Methods:**

In an approximated stepped-wedge wait-list design randomized controlled trial, 277 community-dwelling villagers with schizophrenia were assigned 1:1 in phase 1 into intervention and wait-list control groups. The intervention group received (1) lay health supporters (medication or care supervisors), (2) e-platform (mobile-texting reminders and education message) access, (3) a token gift for positive behavioral changes, and (4) integration with the existing government community-mental health program (the 686 Program) while the wait-listed control group initially only received the 686 Program. Subsequently (in the extended period), both groups received the LEAN intervention plus the 686 Program. The primary outcome was antipsychotic medication adherence (percentage of dosages taken over the past month assessed by unannounced home-based pill counts). The secondary outcomes were symptoms measured during visits to 686 Program psychiatrists using the Clinical Global Impression scale for schizophrenia and functioning measured by trained student assessors using the World Health Organization Disability Assessment Schedule 2.0. Other outcomes included data routinely collected in the 686 Program system (refill records, rehospitalization due to schizophrenia, death for any reason, suicide, wandering, and violent behaviors). We used intention-to-treat analysis and missing data were imputed. A generalized estimating equation model was used to assess program effects on antipsychotics medication adherence, symptoms, and functioning.

**Results:**

Antipsychotics medication adherence improved from 0.48 in the control period to 0.58 in the extended intervention period (adjusted mean difference 0.11, 95% CI 0.04 to 0.19; *P*=.004). We also noted an improvement in symptoms (adjusted mean difference –0.26, 95% CI –0.50 to –0.02; *P*=.04; Cohen *d* effect size 0.20) and a reduction in rehospitalization (0.37, 95% CI 0.18 to 0.76; *P*=.007; number-needed-to-treat 8.05, 95% CI 4.61 to 21.41). There was no improvement in functioning (adjusted mean difference 0.02, 95% CI –0.01 to 0.06; *P*=.18; Cohen *d* effect size 0.04).

**Conclusions:**

In an extended implementation, our intervention featuring mobile texting messages and lay health workers in a resource-poor community setting was more effective than the 686 Program alone in improving medication adherence, improving symptoms, and reducing rehospitalization.

**Trial Registration:**

Chinese Clinical Trial Registry; ChiCTR-ICR-15006053 https://tinyurl.com/y5hk8vng

## Introduction

Affecting 0.4% of the population [1], schizophrenia is a common and disabling condition that presents a dire health equity challenge. Schizophrenia can often be effectively controlled with life-long antipsychotic medications [2]. No access or poor adherence to treatment has serious consequences for the patient, family, and society, leading to a higher risk of worsening symptoms, repeated and prolonged hospitalizations, suicide, aggressive conduct, poor quality of life, and reduced functioning [3]. Nearly half of people with schizophrenia in low- and-middle-income countries take less than 70% of prescribed doses [4]. To address these challenges, the World Health Organization Mental Health Gap Action Program recommended a community-based approach whereby community health workers play an active role in schizophrenia assessment, treatment, rehabilitation, and follow-up [5]. Along the same lines, China rolled out its National Continuing Management and Intervention Program for Psychoses, known as the 686 Program, in 2005 [6,7]. The program covered 5,810,000 people with psychoses across China in 2017 [8] and has become part of China’s “integrated public mental health service [8,9].” However, even though the program provides free medication to individuals with low-incomes, less than 40% of program enrollees routinely adhered to their antipsychotic dosage [7].

Mobile texting or SMS has been successfully used to improve schizophrenia—particularly as a way to improve medication adherence—however, evidence of effectiveness was based on short-term interventions (mostly less than 6 months) or short-term follow-ups and in high-income settings [10-14]. It is important to explore the long-term effect of mobile health interventions because, while longer implementations are able to better shape new behavior and allow sufficient time for effects on functioning and symptoms to occur [15], participants eventually lose interest in program participation. For real-world practicality and sustainability, it is thus critical to examine the effects of the extended implementation of mobile texting interventions.

Initially, we conducted a wait-list randomized controlled study that tested the effectiveness of LEAN, a community-based solution that consists of mobile-texting for medication reminders, relapse monitoring, and education and also a lay health supporter selected from the patient families to help with patient care [16,17]. In this study, we hypothesized that the extended implementation of LEAN (1) would maintain the effects on medication adherence shown in the initial phase, and (2) would begin to show effects on symptoms and functioning that were initially absent.

## Methods

### Protocol

Details of the study design (including sample size calculation and participant randomization), methods, and analysis plan have previously been published as a study protocol [16].

### Data

Data will be freely available upon request after the publication of this manuscript.

### Trial Design

**Figure 1:**
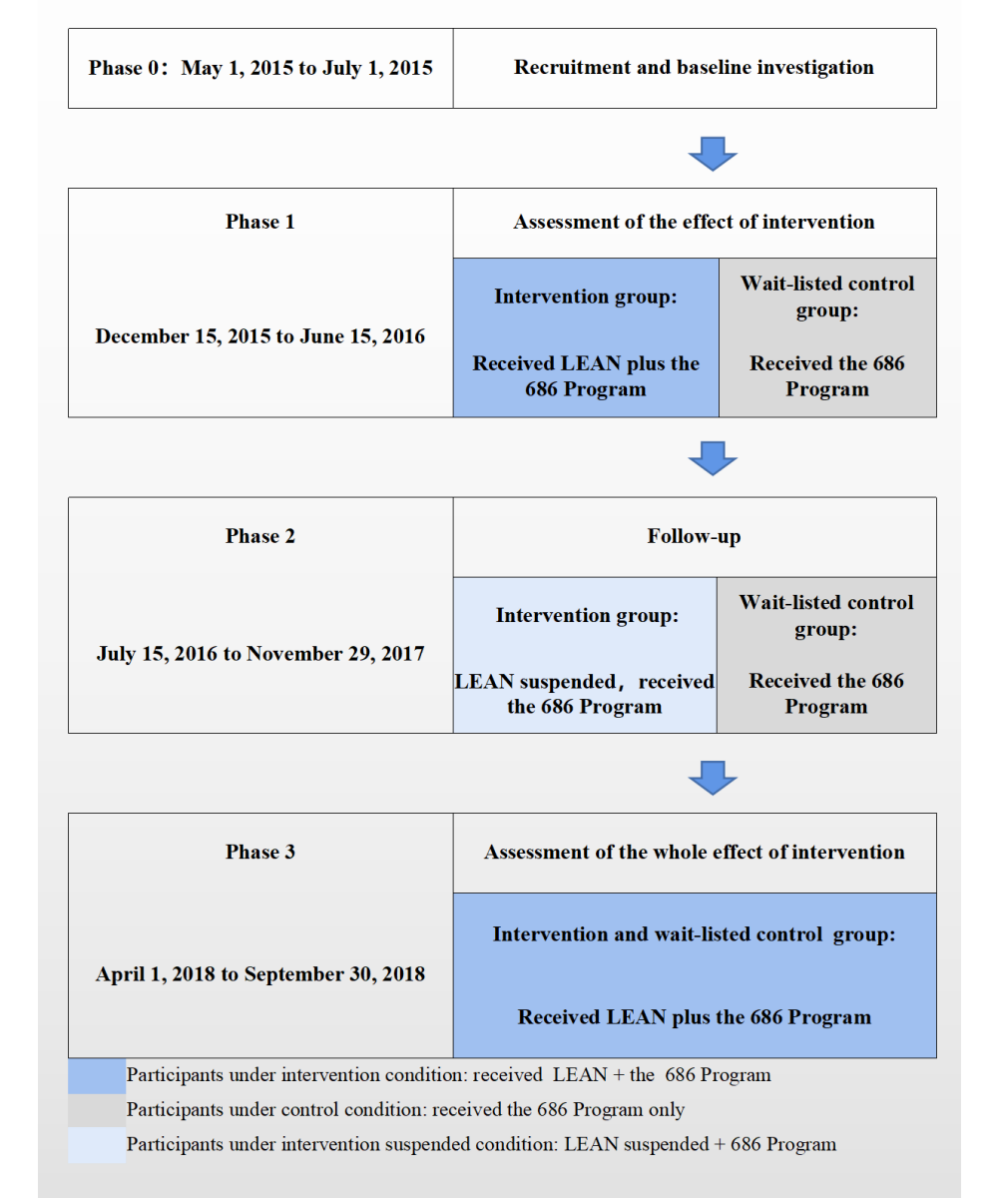
Trial design.

Because of the initial study design [16], in which we employed a wait-listed control group that would receive the intervention once it was proven to be effective, it allowed a 2-period intervention trial similar to a stepped-wedge design trial [18]. The project was executed in 3 phases (Figure 1): phase 1 was from December 15, 2015 to June 15, 2016 during which only the intervention group received LEAN for 6 months; phase 2 (blank phase) consisted of a data analysis period from July 15, 2016 to November 29, 2017 during which neither group received LEAN; and phase 3 was the formal extended trial from April 1, 2018 to September 30, 2018 during which both of the original groups received LEAN for 6 months. The results of phase 1 have been published [17]. 

### Setting and Participants

The trial was conducted in the communities of 9 rural townships of Liuyang municipality (population 356,900) in the Hunan province of China. The patient participants of the program were required to (1) be community-dwelling, (2) be enrollees of the 686 Program, (3) have a primary diagnosis of schizophrenia according to the International Statistical Classification of Diseases, Tenth Revision [19] (diagnosis reconfirmed by the 686 Program psychiatrists while they were enrolled), (4) be on oral psychotropic medication, and (5) be residents of 1 of the 9 rural townships. Patients were excluded if they (1) were hospitalized due to schizophrenia at the time of recruitment (our approach was community-based), or (2) had missed 3 immediate consecutive past drug refills (in this case, they had de facto dropped out of the 686 Program), or (3) were physically incapable of using voice and text messaging (hearing or vision impairment prevented the use of our intervention) [16]. The trial participants were selected by simple random sampling from the 686 Program registry that included almost all known residents of Liuyang diagnosed with schizophrenia.

### Procedures

The development process of our intervention LEAN was described in detail in our earlier paper with phase 1 results [17]. In summary, we used the Health Belief Theory [20-22] and empirical evidence to guide the development of the prototype LEAN intervention, which was refined and finalized through trial and error in the pilot. LEAN includes 4 elements: (1) lay health supporters (often a designated family member to monitor patient medication, side effects, and relapses, and urgent care), (2) e-platform (a texting system for medication reminders, health education, and relapse monitoring), (3) token gifts to encourage behavioral improvement, and (4) integration of the texting with the existing health system to enable collaborative care. Following the same procedure [16], 4 masters’ students in public health produced educational and reminder text messages, which were reviewed by a senior psychiatrist. Frequency and timing of the messages were determined after multiple rounds of consultation with the patients and their families: medication reminders at 7 PM daily, educational messages at 9 AM every other day (these had been sent every day in phase 1), and relapse-monitoring messages monthly. In phase 3, all patients and their lay health supports received the message if they had a cellphone; however, in phase 1, only patients and their lay health supports in the intervention group received the message.

In phase 1, trainers had used one-on-one *demonstration-imitation* to teach patients and lay health supporters in the intervention arm how to use a cellphone to read and reply to the texted messages [23]. Lay health supporters were also trained how to read and reply to relapse-monitoring messages in a group training session in each program township. Lay health supporters were trained to remind the patient to take their medication and report signs of relapse. For the extended intervention, during phase 2, program staff performed the same one-on-one training again during their visits to the patients’ homes. Before the extended intervention was formally implemented, we conducted a 1-week pilot by sending messages to guide participants to practice reading and replying.

Both the initial intervention and control groups received the 686 Program. In Liuyang, 2 internist-transformed psychiatrists along with several staff members traveled with medication supplies to each township’s health center at a fixed date every 2 months, providing patients with a brief consultation and medication adjustment and refills. The township’s mental health administrators (generally public health professionals) informed the patients and their family by phone to meet the traveling psychiatrists and worked with the village doctors (paramedics with rudimentary medical training) to provide yearly physical exams, assessment of risk level, ≥4 home visits throughout the year, health education, and urgent care.

### Outcomes

We continued to track the outcomes specified and detailed in our published protocol [16]. We collected the primary and secondary outcomes in 2 home visits conducted in phase 2 from November 24 to 29, 2017, and from December 24 to 29, 2017, respectively. We again conducted home-based visits from July 19 to 24 and August 19 to 24 in 2018 in phase 3. The primary outcome was antipsychotic medication adherence using the proportion of doses taken in the past month (*number of the first count* – *number of the second count* + *number of additional pills obtained* – *number of pills discarded*) / (*number of pills prescribed*) assessed by unannounced home-based pill-counts. If 2 or more kinds of antipsychotic medications were taken, we used the combined total number of tablets of the medications required by the prescription as the denominator and the actual number of consumed pills as numerator to calculate adherence. Two counts (30 days apart) were necessary to calculate the pills taken over 1 month: the first at the beginning and the second at the end of the last month of each phase. The numbers of pills prescribed were extracted from the 686 Program system. Assessors followed a standard protocol to ask family members and patients about the number of pills additionally purchased and deliverable discarded. The count was considered unannounced since the participants consented to the count but were unaware of the specific timing of the count. Besides, medication refill adherence was assessed (*number of refills required* / *number of refills conducted over past 6 months*), and other self-reported adherence measures were assessed with the Brief Adherence Rating Scale (BARS) [24] and the Drug Attitude Inventory-10 (DAI-10) [25,26] during each home-based interview. Secondary outcomes were patient functioning and patient symptoms assessed with the World Health Organization Disability Assessment Schedule 2.0 (WHO DAS) [27,28] and the Clinical Global Impression in Schizophrenia measure (CGI) [29], respectively.

Trained public health, nursing, and health management students performed the home-based visits. If the patients could communicate with the assessors, the results of BARS, DAI-10, and WHO DAS were reported by the patients, otherwise, the BARS and WHO DAS were reported by their lay health supports. As part of the process evaluation, the students also assessed user experience at the home visits with questionnaires. When 686 Program psychiatrists conducted routine visits to each town at the end of each phase, they performed symptom evaluations using CGI.

We also collected baseline information on medication side effects, substance use, and family supervision when taking medicine since they are strong predictors of adherence that have been empirically suggested by other studies [30]. Medication side effects assessed with the self-reported Glasgow Antipsychotic Side-effect Scale [31]; smoking and alcohol use were also self-reported.

We also extracted routinely collected outcomes from the 686 Program system including rehospitalization due to schizophrenia, death for any reason, suicide, wandering, and violence for the second intervention period, which were verified during the home-base interview. These outcomes are recorded by mental health administrators when they routinely interviewed participants at home or by phone every 3 months. We also captured the frequency that text recipients responded through the texting system log and obtained program-cost information for various program operation channels.

### Statistical Methods

Analyses for phase 3 approximated those for a stepped-wedge randomized controlled design. In a stepped-wedge design, all groups received randomized orders to enter the intervention in sequence. Thus, we no longer used the term intervention or control groups but intervention and control periods instead [32]. The stepped-wedge design maintained the nature of randomization and had the advantage of not only using 2 groups for cross-sectional analysis but also before-and-after comparison of the same group for longitudinal analysis.

We implemented a generalized estimating equation model [33-36] (gee, version 4.8; R, version 3.5.3) in all analyses and used *link identity* for continuous outcomes and logit for binary outcomes and assumed an exchangeable correlation structure. The main results of phase 3 were compared in 2 periods (ie, the intervention and control periods). Statistical significance was set at *P*＜.05.

The analysis of primary outcome (adherence) adjusted for the empirically suggested and prespecified baseline predictors, which were the same as those in phase 1 analysis included baseline adherence, the overall severity of illness, negative symptoms, functioning, substance use, medication side effects, and family supervision. Analyses of the secondary outcomes (WHO DAS and CGI severity of illness and CGI degree of change) were adjusted by their baselines. We used the same analysis models and the covariate adjustment for 2 prespecified subgroups—a group demonstrating baseline nonadherence (missing any of the previous 6 refills was considered nonadherence) and a group with low baseline functioning (cut-off ≤0.22). In extended analyses, we also explored whether the phase 3 intervention led to additional improvement among people who received the intervention in phase 1.

We performed several sensitivity analyses. We compared the results of the program effects on adherence, functioning, and symptoms with a raw analysis, the unadjusted analysis with data imputation for the missing data, and adjusted analysis with covariates and data imputation for the missing data. Meanwhile, for phase 3 analysis, since no one received the intervention during phase 2, we performed a sensitivity analysis by including phase 2 data, coding the intervention group for this phase as 1 and the wait-listed control group as 0. We analyzed antipsychotic medication adherence as a continuous variable, and we also explored the sensitivity of dichotomizing adherence cut-off points of 0.70, 0.80, and 0.90.

We used intention-to-treat analysis [37] for all participants for primary and secondary outcomes. We also conducted a per-protocol analysis. The fully conditional specification multiple imputation method was used to impute missing data [38]. To enable cross-study comparisons, we calculated the program effect size using Cohen *d* [39].

### Ethics and Dissemination

The study obtained institution review board approval from the University of Washington (49464 G) and Central South University (CTXY-150002-6). All patient participants and their lay health supporters provided written informed consent.

## Results

### Participants

**Figure 2:**
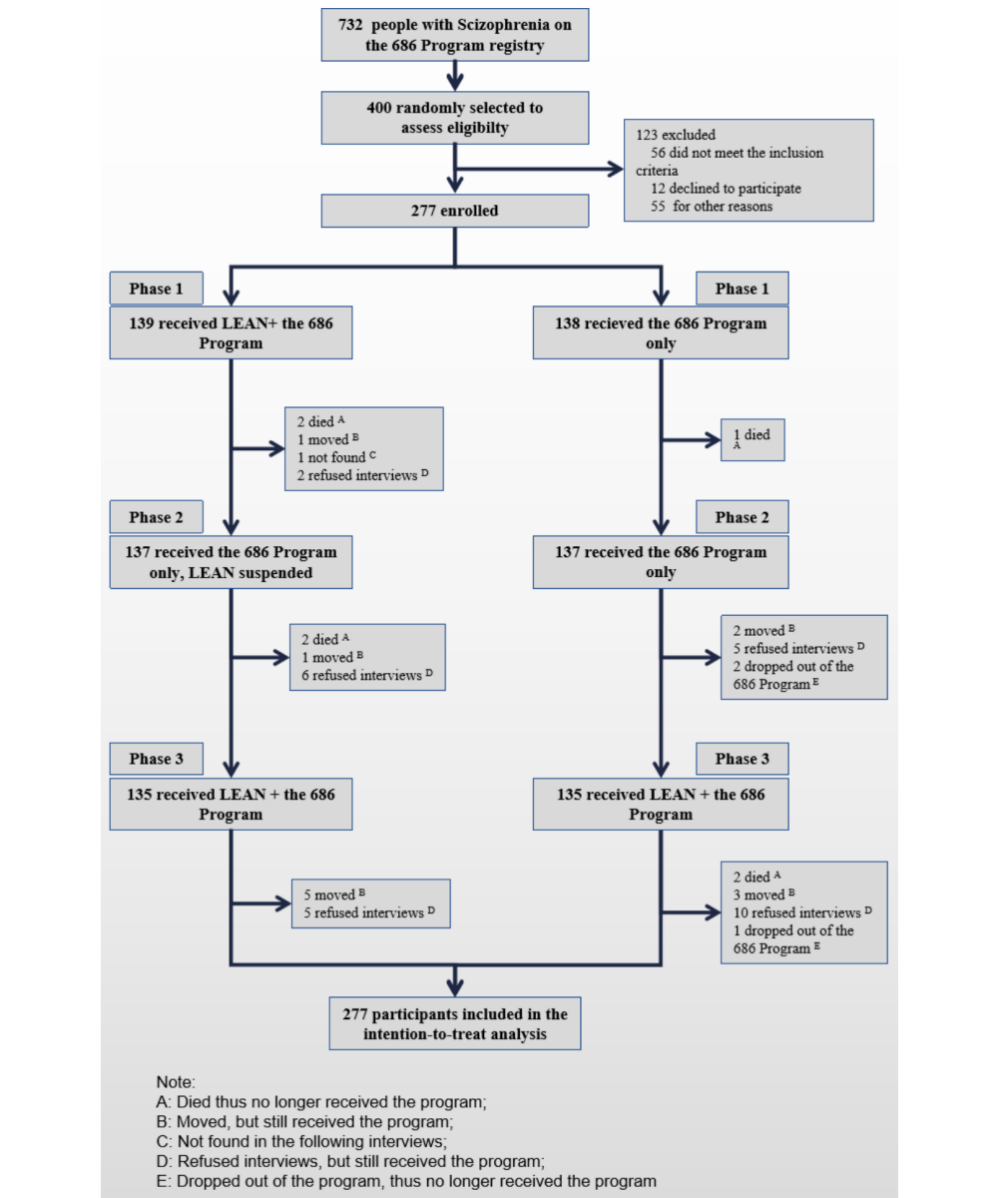
Participant flowchart.

In phase 1, there were 277 patient participants (intervention, n=139; wait-listed control group, n=138) (Figure 2). Due to the closed-cohort design, participants assessed in different periods remained the same. The baseline characteristics were well balanced between the 2 groups [17]. There were 244 participates successfully followed in phase 3. In phase 1, each patient participant in the intervention group had a designated lay health supporter, and in phase 3, the lay health supporters in the original intervention group continued to take the same responsibility. Each of the participants in the original control group also received a lay health supporter. The supporters were mainly family members (129/138), and the rest of the other supporters were community volunteers or village doctors.

### Retention and Outcomes

At the end of phase 3, we captured the information of 271/277 (97.8%) participants for medication refill adherence, 164/277 (59.2%) for pill-count adherence, 231/277 (83.4%) for functioning (WHO DAS), and 247/277 (89.17%) for symptoms. Analyses of patterns of missing data and the results of multiple imputation are presented in Multimedia Appendix 1.

### Adherence

**Figure 3:**
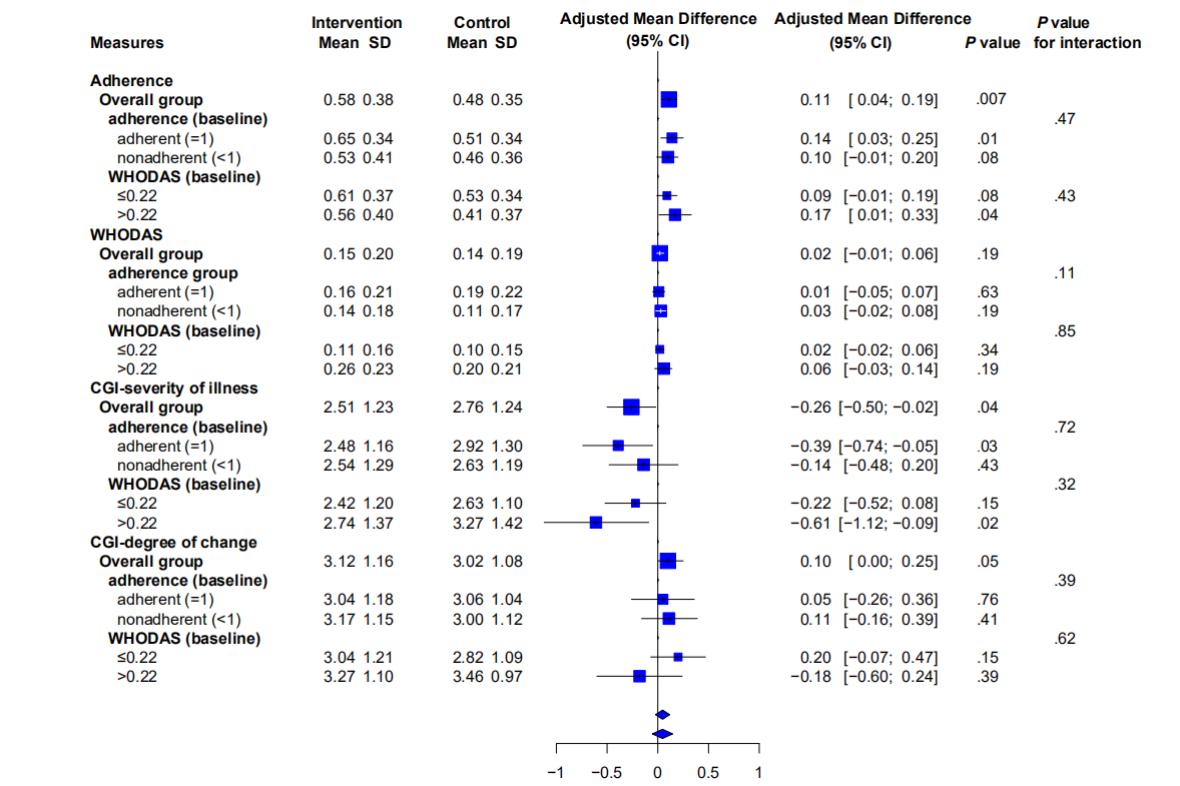
Subgroup analysis. CGI: Clinical Global Impression; WHODAS: World Health Organization Disability Assessment Schedule.

In phase 3, adherence increased from 0.48 in the control period (ie, control arm in phase 1) to 0.58 in the intervention periods (ie, intervention arm in phase 1 and both arms in phase 3). Model-based adjusted analysis showed the increase in antipsychotic adherence remained statistically significant (adjusted mean difference 0.11, 95% CI 0.04 to 0.19; *P=*.004; Cohen *d* effect size 0.28; Table 1). Subgroup analyses suggested that the participants with better adherence and poor functioning at baseline presented better adherence at the end of phase 3 (Figure 3). Distribution of adherence between the intervention and the control period are shown in Multimedia Appendix 2, and the reasons for zero adherence are explored in Multimedia Appendix 3.

**Table 1:** Results.

Measures				Intervention periods, mean (SD)		Control periods, mean (SD)		Mean difference (95% CI)		*P* value
**Adherence outcomes**										
	Pill-count assessment^a^			0.58 (0.38)		0.48 (0.35)		0.11 (0.04, 0.19)		.004
	**Other**									
		Medication refill assessment	0.77 (0.35)		0.76 (0.34)		–0.01 (–0.06, 0.04)		.70	
		DAI-10^b^	0.67 (0.23)		0.67 (0.22)		0.01 (–0.04, 0.06)		.73	
		BARS^c^	0.71 (0.20)		0.68 (0.23)		0.04 (–0.01, 0.08)		.13	
**Secondary outcomes**										
	WHO^d^ DAS^a,e^			0.15 (0.19)		0.15 (0.19)		0.02 (–0.01, 0.06)		.19
	**CGI^f^ severity of illness^a,g^**			2.51 (1.23)		2.76 (1.24)		–0.26 (–0.50, 0.02)		.04
		Negative	2.41 (1.17)		2.98 (1.43)		–0.47 (–0.76, 0.19)		.001	
		Positive	2.42 (1.19)		2.67 (1.55)		–0.47 (–0.76, 0.17)		.002	
		Depression	1.9 (1.01)		2.11 (1.26)		–0.21 (–0.44, 0.01)		.07	
		Cognition	2.44 (1.19)		2.85 (1.44)		–0.50 (–0.79, 0.21)		<.001	
	CGI degree of change^a,h^			3.12 (1.16)		3.02 (1.08)		0.10 (0.00, 0.25)		.05
	**686 Program**									
		Rehospitalization due to schizophrenia	43 (11.1)		25 (19.5)		0.58 (0.37, 0.91)		.02	
		Death^i^	0 (1.0)		3 (2.2)		N/A		N/A	
		Suicide^i^	0 (0.0)		1 (0.0)		N/A		N/A	
		Wandering	7 (1.7)		2 (1.5)		0.98 (0.23, 4.13)		.98	
		Hurting people or smashing objects	2 (0.4)		6 (4.5)		0.11 (0.02, 0.54)		.006	
		Getting into trouble	0 (0.0)		0 (0.0)		0.11 (0.04, 0.19)		N/A	
		Self-harm	0 (0.0)		0 (0.0)		–0.01 (–0.06, 0.04)		N/A	

^a^Adjusted for baseline covariates with imputation for missing data.

^b^DAI: Drug Attitude Inventory. The score was rescaled as 0 to 1.

^c^BARS: Brief Adherence Rating Scale.

^d^WHO: World Health Organization.

^e^DAS: Disability Assessment Schedule (range 0 to 1).

^f^CGI: Clinical Global Impression.

^g^Higher scores indicate worse symptoms (range 1 to 7).

^h^Higher scores indicate less change (range 1 to 7).

^i^The numbers are for phase 3 only.

### Functioning

There were 31.8% (75/236) WHO DAS questionnaires completed by lay health supporters on behalf of patient participants. Interviewees were the patient’s parents (31/75), spouses (28/75), offspring (8/75), siblings (6/75), and others (2/75) (Multimedia Appendix 4). At the end of phase 3, we observed no improvement in functioning (Table 1). For the raw analysis, the WHO DAS score changed from 0.14 under the control period to 0.15 under the intervention periods; our model-based analysis showed no difference (adjusted mean difference 0.02, 95% CI –0.01 to 0.06; *P=*.18; Cohen *d* effect size 0.04; Table 1). Neither did we note any improvement in the severity of functioning for the prespecified subgroups (Figure 3).

### Symptoms

The severity of illness decreased (CGI score 2.76 in the control period to 2.51 in the intervention periods (model-based adjusted mean difference –0.26, 95% CI –0.50 to –0.02; *P=*.04; Cohen *d* effect size 0.20). All domains of the CGI (negative: *P*=.001, positive: *P*=.002, cognition: *P*<.001) except depression (*P*=.07) showed significant improvements. Subgroup analyses suggested that the baseline adherence group and poor functioning group presented symptom improvement at the end of phase 3 as well (Figure 3).

### Other Outcomes

The incidence of rehospitalization due to schizophrenia was 19.5% (25/128) in the control period versus 11.1% (43/387) in the intervention periods (relative risk 0.58, 95% CI 0.37 to 0.91, *P*=.02, number-needed-to-treat 12.25 95%CI 6.36 to 166.49; Table 1).

There was a significant difference in violence (hurting people or smashing objects) (*P*=.006). However, there were no significant differences in wandering (*P*=.98) and in other outcomes captured in the 686 Program. Eight patients died during the entire follow-up including 2 patients who committed suicide (all-cause mortality 28.88/1000 person-year).

### Extended Analyses and Sensitivity Analyses

Through our extended analyses, we found that the phase 3 intervention did not result in any additional improvement in adherence (mean difference –0.034, 95% CI –0.141 to 0.073; *P*=.531) and functioning (mean difference –0.84, 95% CI –0.626 to 0.458; *P=*.084) among participants who received the intervention in phase 1. However, their symptoms were further improved in phase 3 (mean difference –0.407, 95% CI –0.713 to –0.101; *P=*.009).

Sensitivity analyses (see Multimedia Appendix 5) showed that the results were not sensitive to the different analytical methods used. The effects of the alternative analytical approaches were almost identical to that of our primary analysis, probably mainly due to the possible reasons that (1) missing data were possibly missing at random, and (2) few participant-lay health supporter pairs did not receive the intervention (14/270, 5.2%), and the exclusion of these samples had little effect on the results of the analysis.

### Process Indicators

Process indicators including the process of texting, user experiences, and cost information are presented in Multimedia Appendix 6. We also present example text messages in Multimedia Appendix 6.

## Discussion

### Principal Results

In this stepped wedge–like randomized controlled trial, we found that a long-term intervention of texting for individuals with schizophrenia and their lay health supporters improved pill count–based adherence to antipsychotic medication from 0.48 during the control period to 0.58 during the intervention period, reduced CGI severity of illness score from 2.76 to 2.51, and reduced rehospitalization from 19.7% to 11.8%.

As of December 1, 2019, we identified 6 randomized controlled trials of mobile texting to improve care for people with schizophrenia [10-14,40]. The 4 trials that had less than 6 months of intervention all presented positive effects of the intervention [10-14,40]. However, two 12-month interventions showed no difference between the intervention and control groups [13,40]. All of these previous trials were conducted in high-income countries. In the LEAN trial, mobile texting improved adherence with a Cohen *d* effect size of 0.28, smaller than that of Montes et al’s study [12], which used patient self-reported adherence measured with the Morisky Green Adherence Questionnaire, but larger than that of Beebe et al’s study [14], which also used in-home pill-count adherence (Multimedia Appendix 7). We suspect our texting of both the patients and their lay health supporters (mostly family members) was a major contributor to LEAN’s relative superiority in the long-term implementation. Our texted medication reminders, health education, and relapse monitoring may have provided tools and cues for the family to take more active and timely action in supervising medications. Despite LEAN’s effect, we should also note that there seemed to be a decline in improving adherence in the long-term intervention (0.48 in control to 0.61 in phase 1 [17] versus 0.48 to 0.58 in phase 3). Our analyses suggested two possible explanations. First, because these individuals with schizophrenia lack awareness of the severity of the disease, more patients stopped taking their medication in phase 3. This may be caused by the intervention not helping these participants to develop a belief and hence sustainable behavior to keep taking medicine. Second, we observed a sign of participants’ fatigue toward the program, as both participant's satisfaction toward LEAN and the family response rates decreased from phase 1 to phase 3, (Multimedia Appendix 6, Table S2). Välimäki et al [40] also reported fatigue in their study. Therefore, to sustain mobile health interventions for people with schizophrenia, timely adjustments to the program may be required to counter the possible decline in participants’ satisfaction.

We expected that increased antipsychotic medication adherence would translate into improved symptoms and functioning in the long-term. However, a possible ceiling effect may have prevented further improvements in functioning. Likely due to the 686 Program that both the intervention and control groups received, LEAN participants at baseline had much better functioning (WHO DAS score 0.18) than other groups of people with schizophrenia of similar ethnicity and culture have shown (WHO DAS score ranging from 0.29 [41] to 0.64 [42]). However, despite the overall mild symptoms of LEAN participants, an improvement in symptoms (reduction of CGI severity of illness score from 2.76 to 2.51) was still observed at the end of phase 3 but not in phase 1. This may suggest that over an extended time the enhancement in medication adherence may gradually result in symptom reduction. Furthermore, LEAN’s effect on symptoms was in concordance with the observed large reduction in rehospitalization due to schizophrenia.

In extended analyses, we found that the phase 3 intervention led to additional improvement in symptoms but not in adherence and functioning among people with schizophrenia who received the intervention in phase 1. We suspect that further improvements in adherence and functioning were difficult due to a ceiling effect. All participants had received the 686 Program, and thus a basic level of function had been maintained. However, changes in symptoms may be more sensitive to changes in adherence over a long time.

### Limitations

We should note several limitations of the LEAN trial. First, we did not take a systematic approach in assessing the reasons for the participants discontinuing and continuing antipsychotic medications. Future trials may consider using scales such as the Antipsychotic Discontinuation Questionnaire [43-45]. Second, we obtained the incidence of relapse (defined as the marked occurrence of symptoms) by in-person interviewing of the family carers in phase 1 and by self-reported relapse from people with schizophrenia or their lay health supporters through their response to our relapse monitoring messages in phase 3. We felt more objective and defined measures for relapse should be used for future studies. Third, the blank phase (phase 2) when neither the intervention nor the control groups received LEAN created an aberration to the standard stepped-wedge design, although we included this phase as a covariate in the secondary model. Fourth, in phase 3, 34.1% (92/270) patient participants and 17.8% (48/270) lay health supporters were not equipped with a phone; however, there were a total of 5.2% (14/270) pairs (of patient participants and lay health supporters) not equipped with a phone, either not having a phone or not having replaced a damaged, malfunctioning, or lost phone in phase 1. Fifth, our intervention was an integrated package that combined 4 program elements, of which 2 critical components were the use of lay health supporters and text messages. We are not able to determine the individual contribution of either component to the program’s effect. In our study, we did not systematically collect information about the depth of the involvement of the lay health supporters. We were unable to quantitatively isolate the effect of the role played by the lay health supporters in the overall intervention package. However, we did track the satisfaction level of the supporters and found out that there was a decreasing trend in overall satisfaction near the end of our intervention. It showed that 80.0% (100/125) lay health supporters surveyed were satisfied with the intervention, and 78.4% (98/125) expressed their willingness to remain in phase 3 (see Multimedia Appendix 6, Table S2). Texting system records showed that 47.3% (105/222) of family members responded to the texted reminders. Future studies may consider multiphase optimization strategy and sequential multiple assignment randomized trial designs to fully address those issues [46,47]. Besides, LEAN used daily text reminders but the optimal frequency was not tested. The use of multiphase optimization and sequential multiple assignment randomized trial designs can also address this issue well. Finally, although we used a rigorous method of adherence assessment through unannounced home-based pill-counts, we could not completely address the issue of participants deliberately discarding pills. However, this should not affect our program impact evaluation as the behavior may occur in both the control and intervention periods. Also, we believe our method of assessing adherence is best suited for this study—other methods, such as an electronic cap, may remind people to take medications and thus interfere in the unbiased assessment of adherence [48].

Due to the complexity of analysis and the different hypotheses tested, we decided to cover the details of the results of phase 2 in a separate manuscript that will primarily explore the maintenance of the effects after the withdrawal of the intervention.

### Comparison With Prior Work

Universal health coverage is not attainable without the coverage for mental health. China’s 686 Program provides valuable experiences for other low-resource settings to develop community-based care at scale. LEAN further enabled and motivated family carers and patients with an easy-to-implement and low-cost texting system. The use of mobile texting for mental health is perhaps no longer considered novel. However, few trials have attempted to test its effectiveness in long-term implementation. LEAN spanned over 2 years with 1 year of dedicated texting and demonstrated overall effectiveness in improving medication adherence, symptoms, and rehospitalization. Although LEAN was built upon China’s 686 Program, many elements of LEAN should be useful for other low-resource settings with or without an existing community-based program. Implications of LEAN results to other low-resources settings should note the following. First, the overall adherence to antipsychotic medication at the endpoint remained low at 0.57 (SD 0.40) despite free medication from the 686 Program and the LEAN efforts. Long-acting injectable antipsychotic medications, in treating schizophrenia, were cost-effective [49] and reduced relapse [50] but have not been widely accepted by clinicians and families with schizophrenia in China [51]. Second, formal health workers in China and other low-resource settings are often overextended, so task-shifting is important such as engaging the self-motivated family members in monitoring medication, side effects, and relapse. LEAN provides a relatively low-cost approach to assist the family carers in their roles. In our study, the majority of lay health supporters were family members who lived with the patient participants (258/277); they reminded them to take their medication and monitored signs of side-effects and relapses. Third, SMS played a very important role in connecting the patient participants, their families, medical staff, and the health care system. It created a way of communication and timely exchange between the family members and the medical staff. It may have also reduced the isolation of the patient participants and their family members. Even though user satisfaction with LEAN remained high throughout the program, it dropped considerably from 98.4% (62/63) to 67.4% (66/98) for patients and 100% (77/77) to 80.0% (100/125) for the lay health supporters from phase 1 to phase 3 (Multimedia Appendix 6, Table S2), In future studies, the frequency, content, and timing of texting should be optimized for each set to reduce user fatigue [52]. Finally, considering the reduced cognition of some people with schizophrenia, we used the most rudimentary model of text messaging without individual tailoring and smartphone-based apps. Other settings should fully consider the cost, feasibility, and acceptability in their community and family context to determine the optimal program mode.

### Conclusions

In our study, we found that our intervention featuring mobile texting messages and lay health workers in a resource-poor community setting was more effective in improving medication adherence, symptoms, and rehospitalization than the 686 Program alone in long-term implementation. The experiences of LEAN can potentially be widely applicable to improve medication adherence of other chronic diseases in other resource-poor settings.

## Appendix

Multimedia Appendix 1 The treatment of missing data.

Multimedia Appendix 2 Frequency of participants with different pill-count adherence in phase 1 and phase 3.

Multimedia Appendix 3 Reasons for discontinuing.

Multimedia Appendix 4 Relationship between WHODAS interviewees and patients.

Multimedia Appendix 5 Sensitivity analyses.

Multimedia Appendix 6 Process indicators.

Multimedia Appendix 7 Cohen d effect size of the primary outcome in other research.

## Abbreviations

BARS: Brief Adherence Rating Scale
CGI: Clinical Global Impression
DAI-10: Drug Attitude Inventory-10
DAS: Disability Assessment Schedule
SMS: short message service
WHO: World Health Organization

## References

[1] McGrath J, Saha S, Chant D, Welham J (2008). Schizophrenia: a concise overview of incidence, prevalence, and mortality. Epidemiol Rev.

[2] Leucht S, Cipriani A, Spineli L, Mavridis D, Örey D, Richter F, Samara M, Barbui C, Engel RR, Geddes JR, Kissling W, Stapf MP, Lässig B, Salanti G, Davis JM (2013). Comparative efficacy and tolerability of 15 antipsychotic drugs in schizophrenia: a multiple-treatments meta-analysis. The Lancet.

[3] Iasevoli F, Giordano S, Balletta R, Latte G, Formato MV, Prinzivalli E, De Berardis D, Tomasetti C, de Bartolomeis A (2016). Treatment resistant schizophrenia is associated with the worst community functioning among severely-ill highly-disabling psychiatric conditions and is the most relevant predictor of poorer achievements in functional milestones. Prog Neuropsychopharmacol Biol Psychiatry.

[4] Goff DC, Hill M, Freudenreich O (2010). Strategies for improving treatment adherence in schizophrenia and schizoaffective disorder. J Clin Psychiatry.

[5] mhGAP Mental Health Gap Action Programme—scaling up care for mental, neurological and substance use disorders. World Health Organization.

[6] Good BJ, Good MD (2012). Significance of the 686 Program for China and for global mental health. Shanghai Arch Psychiatry.

[7] Kang HM, Guan LL, Wang X, Wu XM (2015). Economic condition，basic medical insurance and medication among patients with severe mental illness in 31 demonstration cities for“686 program”implementation [In Chinese]. Chin J Public Health.

[8] Wu XM, Ma M, Wang X, Zhang WF (2019). Management and services for psychosis in the People?S Republic of China in 2017[In Chinese]. China Journal Psychiatry.

[9] Liang D, Mays VM, Hwang W (2018). Integrated mental health services in China: challenges and planning for the future. Health Policy Plan.

[10] Pijnenborg GHM, Withaar FK, Brouwer WH, Timmerman ME, van DBRJ, Evans JJ (2010). The efficacy of SMS text messages to compensate for the effects of cognitive impairments in schizophrenia. Br J Clin Psychol.

[11] Granholm E, Ben-Zeev D, Link PC, Bradshaw KR, Holden JL (2012). Mobile Assessment and Treatment for Schizophrenia (MATS): a pilot trial of an interactive text-messaging intervention for medication adherence, socialization, and auditory hallucinations. Schizophr Bull.

[12] Montes JM, Medina E, Gomez-Beneyto M, Maurino J (2012). A short message service (SMS)-based strategy for enhancing adherence to antipsychotic medication in schizophrenia. Psychiatry Res.

[13] Španiel F, Hrdlička J, Novák T, Kožený J, Höschl C, Mohr P, Motlová LB (2012). Effectiveness of the information technology-aided program of relapse prevention in schizophrenia (ITAREPS): a randomized, controlled, double-blind study. J Psychiatr Pract.

[14] Beebe L, Smith KD, Phillips C (2014). A comparison of telephone and texting interventions for persons with schizophrenia spectrum disorders. Issues Ment Health Nurs.

[15] Wiersma D, Wanderling J, Dragomirecka E, Ganev K, Harrison G, An Der Heiden W, Nienhuis F J, Walsh D (2000). Social disability in schizophrenia: its development and prediction over 15 years in incidence cohorts in six European centres. Psychol Med.

[16] Xu DR, Gong W, Caine ED, Xiao S, Hughes JP, Ng M, Simoni J, He H, Smith KL, Brown HS, Gloyd S (2016). Lay health supporters aided by a mobile phone messaging system to improve care of villagers with schizophrenia in Liuyang, China: protocol for a randomised control trial. BMJ Open.

[17] Xu DR, Xiao S, He H, Caine ED, Gloyd S, Simoni J, Hughes JP, Nie J, Lin M, He W, Yuan Y, Gong W (2019). Lay health supporters aided by mobile text messaging to improve adherence, symptoms, and functioning among people with schizophrenia in a resource-poor community in rural China (LEAN): A randomized controlled trial. PLoS Med.

[18] Beard E, Lewis JJ, Copas A, Davey C, Osrin D, Baio G, Thompson JA, Fielding KL, Omar RZ, Ononge S, Hargreaves J, Prost A (2015). Stepped wedge randomised controlled trials: systematic review of studies published between 2010 and 2014. Trials.

[19] The ICD-10 classification of mental and behavioural disorders: clinical descriptions and diagnostic guidelines. World Health Organization.

[20] Becker MH, Maiman LA (1975). Sociobehavioral determinants of compliance with health and medical care recommendations. Med Care.

[21] Rosenstock IM, Strecher VJ, Becker MH (1988). Social learning theory and the Health Belief Model. Health Educ Q.

[22] Finfgeld DL, Wongvatunyu S, Conn VS, Grando VT, Russell CL (2003). Health belief model and reversal theory: a comparative analysis. J Adv Nurs.

[23] Zhao M, Gong WJ, Xu DR, Huang ZP, Hu B (2018). Nie J The Training Effecton Text-messaging Skills of Patients with Schizophreniain rural China and Its Influencing Factors In Chinese. China Journal of Health Psychology.

[24] Byerly MJ, Nakonezny PA, Rush AJ (2008). The Brief Adherence Rating Scale (BARS) validated against electronic monitoring in assessing the antipsychotic medication adherence of outpatients with schizophrenia and schizoaffective disorder. Schizophr Res.

[25] Cheng HL, Yu YW (1997). [Validation of the Chinese version of "the Drug Attitude Inventory"]. Kaohsiung J Med Sci.

[26] Stjernswärd S, Persson K, Nielsen R, Tuninger E, Levander S (2013). A modified Drug Attitude Inventory used in long-term patients in sheltered housing. Eur Neuropsychopharmacol.

[27] Guilera G, Gómez-Benito Juana, Pino O, Rojo JE, Cuesta MJ, Martínez-Arán Anabel, Safont G, Tabarés-Seisdedos Rafael, Vieta E, Bernardo M, Crespo-Facorro B, Franco M, Rejas J (2012). Utility of the World Health Organization Disability Assessment Schedule II in schizophrenia. Schizophr Res.

[28] Sjonnesen K, Bulloch AGM, Williams J, Lavorato D, B Patten Scott (2016). Characterization of disability in Canadians with mental disorders using an abbreviated version of a DSM-5 emerging measure: the 12-Item WHO Disability Assessment Schedule (WHODAS) 2.0. Can J Psychiatry.

[29] Haro JM, Kamath SA, Ochoa S, Novick D, Rele K, Fargas A, Rodríguez M J, Rele R, Orta J, Kharbeng A, Araya S, Gervin M, Alonso J, Mavreas V, Lavrentzou E, Liontos N, Gregor K, Jones PB, SOHO Study Group (2003). The Clinical Global Impression-Schizophrenia scale: a simple instrument to measure the diversity of symptoms present in schizophrenia. Acta Psychiatr Scand Suppl.

[30] Fenton WS, Blyler CR, Heinssen RK (1997). Determinants of medication compliance in schizophrenia: empirical and clinical findings. Schizophr Bull.

[31] Waddell L, Taylor M (2008). A new self-rating scale for detecting atypical or second-generation antipsychotic side effects. J Psychopharmacol.

[32] Hemming K, Haines TP, Chilton PJ, Girling AJ, Lilford RJ (2015). The stepped wedge cluster randomised trial: rationale, design, analysis, and reporting. BMJ.

[33] Hussey MA, Hughes JP (2007). Design and analysis of stepped wedge cluster randomized trials. Contemp Clin Trials.

[34] Barker D, McElduff P, D'Este C, Campbell MJ (2016). Stepped wedge cluster randomised trials: a review of the statistical methodology used and available. BMC Med Res Methodol.

[35] Khadka A, Perales NA, Wei DJ, Gage AD, Haber N, Verguet S, Patenaude B, Fink G (2018). Malaria control across borders: quasi-experimental evidence from the Trans-Kunene malaria initiative (TKMI). Malar J.

[36] Li F, Turner EL, Preisser JS (2018). Sample size determination for GEE analyses of stepped wedge cluster randomized trials. Biometrics.

[37] Hollis S, Campbell F (1999). What is meant by intention to treat analysis? survey of published randomised controlled trials. BMJ.

[38] van Buuren S (2012). Flexible Imputation of Missing Data.

[39] Cohen J (1988). Statistical Power Analysis for the Behavioral Sciences.

[40] Välimäki Maritta, Kannisto KA, Vahlberg T, Hätönen Heli, Adams CE (2017). Short text messages to encourage adherence to medication and follow-up for people with psychosis (Mobile.Net): randomized controlled trial in Finland. J Med Internet Res.

[41] Chen R, Liou T, Miao N, Chang K, Yen C, Liao H, Chi W, Chou K (2020). Using World Health Organization Disability Assessment Schedule 2.0 in people with schizophrenia: a 4-year follow-up. Eur Arch Psychiatry Clin Neurosci.

[42] Dan A, Kumar S, Avasthi A, Grover S (2011). A comparative study on quality of life of patients of schizophrenia with and without depression. Psychiatry Res.

[43] Matza LS, Phillips GA, Revicki DA, Ascher-Svanum H, Kaiser D, Stauffer V, Shorr JM, Kinon BJ (2011). Development of a clinician questionnaire and patient interview to assess reasons for antipsychotic discontinuation. Psychiatry Res.

[44] Matza L, Ascher-Svanum H, Kinon B, Phillips, Revicki, Malley, Palsgrove, Faries, Stauffer, Awad, Keefe, Naber (2012). Validation of a patient interview for assessing reasons for antipsychotic discontinuation and continuation. Patient Prefer Adherence.

[45] Matza LS, Phillips GA, Revicki DA, Ascher-Svanum H, Malley KG, Palsgrove AC, Faries DE, Stauffer V, Kinon BJ, George Awad A, Keefe RS, Naber D (2012). Validation of a clinician questionnaire to assess reasons for antipsychotic discontinuation and continuation among patients with schizophrenia. Psychiatry Res.

[46] Collins LM, Murphy SA, Strecher V (2007). The multiphase optimization strategy (MOST) and the sequential multiple assignment randomized trial (SMART): new methods for more potent eHealth interventions. Am J Prev Med.

[47] Collins LM, Nahum-Shani I, Almirall D (2014). Optimization of behavioral dynamic treatment regimens based on the sequential, multiple assignment, randomized trial (SMART). Clin Trials.

[48] Xu DR, Gong W, Gloyd S, Caine ED, Simoni J, Hughes JP, Xiao S, He W, Dai B, Lin M, Nie J, He H (2018). Measuring adherence to antipsychotic medications for schizophrenia: concordance and validity among a community sample in rural China. Schizophr Res.

[49] Yang L, Li M, Tao L, Zhang M, Nicholl MD, Dong P (2009). Cost-effectiveness of long-acting risperidone injection versus alternative atypical antipsychotic agents in patients with schizophrenia in China. Value Health.

[50] Leucht C, Heres S, Kane JM, Kissling W, Davis JM, Leucht S (2011). Oral versus depot antipsychotic drugs for schizophrenia--a critical systematic review and meta-analysis of randomised long-term trials. Schizophr Res.

[51] Xiang Y, Weng Y, Leung C, Tang W, Ungvari G (2008). Clinical and social correlates with the use of depot antipsychotic drugs in outpatients with schizophrenia in China. Int J Clin Pharmacol Ther.

[52] Berry N, Lobban F, Emsley R, Bucci S (2016). Acceptability of interventions delivered online and through mobile phones for people who experience severe mental health problems: a systematic review. J Med Internet Res.

