# Supplementary material for: Mobile Texting and Lay Health Supporters to Improve Schizophrenia Care in a Resource-Poor Community in Rural China (LEAN Trial): Randomized Controlled Trial Extended Implementation
Source: J Med Internet Res. 2020 Dec 1;22(12):e22631. doi: 10.2196/22631 (PMC7738261; doi:10.2196/22631)
Supplement: Multimedia Appendix 1 [file jmir_v22i12e22631_app1.docx]

# Web appendix

## Appendix 1. The Treatment of Missing Data

We use multiple imputation method to deal with missing data:

In the analysis process, we mainly use the R package mice, gee, norm. (MICE: Multivariate Imputation by Chained Equations). In the multiple imputations, we imputed 10 complete datasets. The GEE model is then applied for each dataset, and the systemization of 10 GEE model results is done with the norm R package. We describe the specific steps below, using the analysis of the effect of the program on medication adherence:

1. Assessing the missing data pattern: We visualized the pattern of missing data for antipsychotics medication adherence to discern any systematic pattern of missing (Figure 1). As we did not find any clear pattern, we assume that data are missing at random(MAR) in our analysis.

2. Selecting the data imputation model: If the variable with missing value is a continuous variable, the predictive mean matching (PMM) method is used; if the variable with missing value is a binary variable, Logistic regression is then used; while for a multi-level categorical missing variable, the polytomous regression is applied.

3. Determining the independent variables used in the imputation model: all other variables except for the dependent variable were used in the imputation model. To improve the accuracy of the imputation, in addition to the variables used for the primary GEE model including the variables of intervention assignment, adherence, functioning, substance use, drug side effects, family supervision, we also used other social-economic and demographic variables such as sex, age, education, income, living alone, duration of illness, symptoms severity etc.in the imputation model.

4. Determining the order of imputation: From left to right: demographic/social-economic information and other variables, the independent variables used in the GEE model, dependent variables used in the GEE model.

5. Ten complete datasets generated were generated: Figure 2 shows the original observed data and the imputed data. The imputed data sets show a similar pattern of the data distribution to the original dataset.

6. The primary GEE model was performed for each of the 10 complete data sets.

7. Using the norm R package to synthesize the results from the 10 GEE models to obtain the overall estimate of the effect of the program.

References: MICE: Multivariate Imputation by Chained Equations in R

| 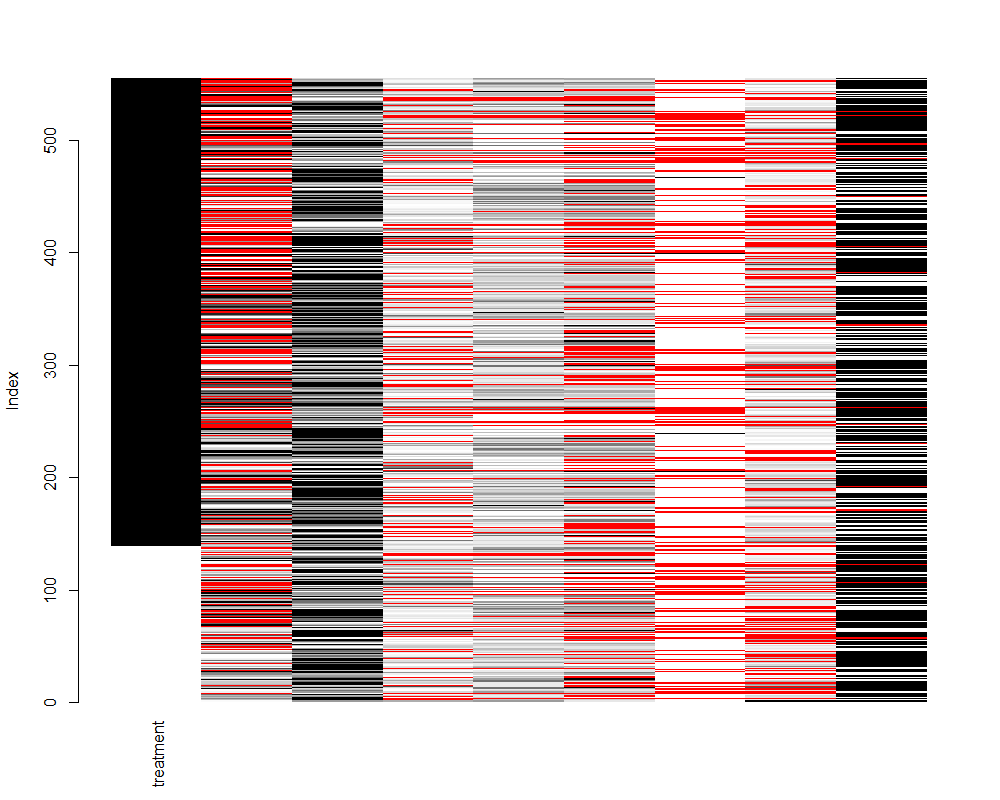  （a） | 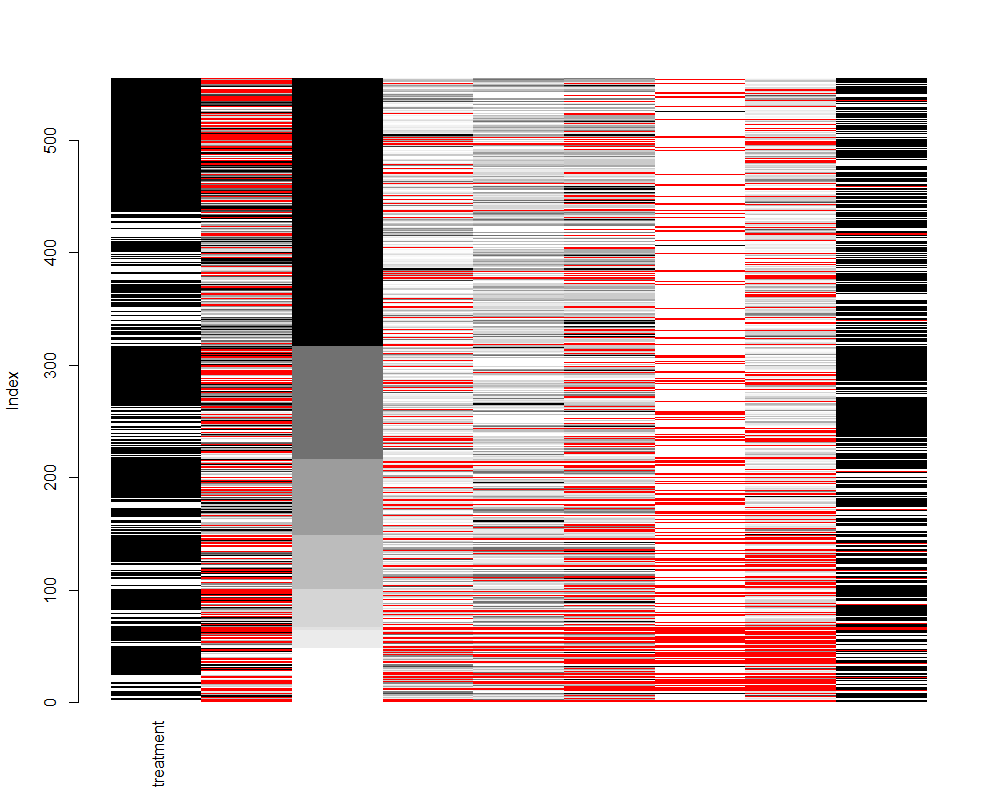  （b） |
| --- | --- |
| 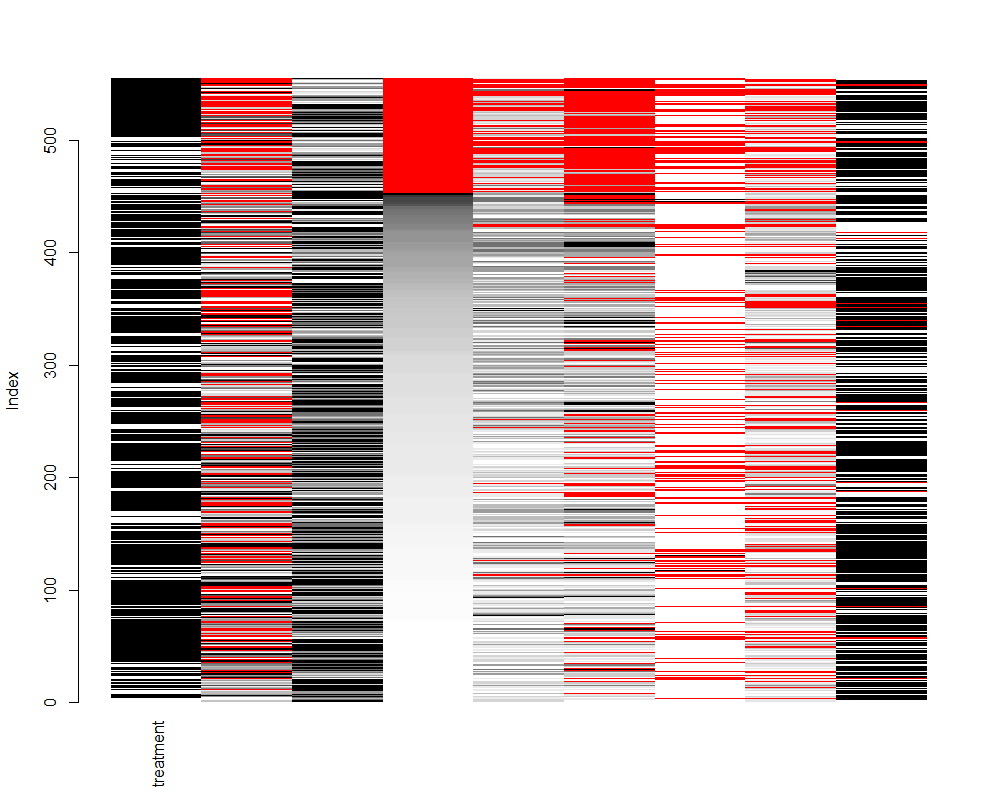  （c） | 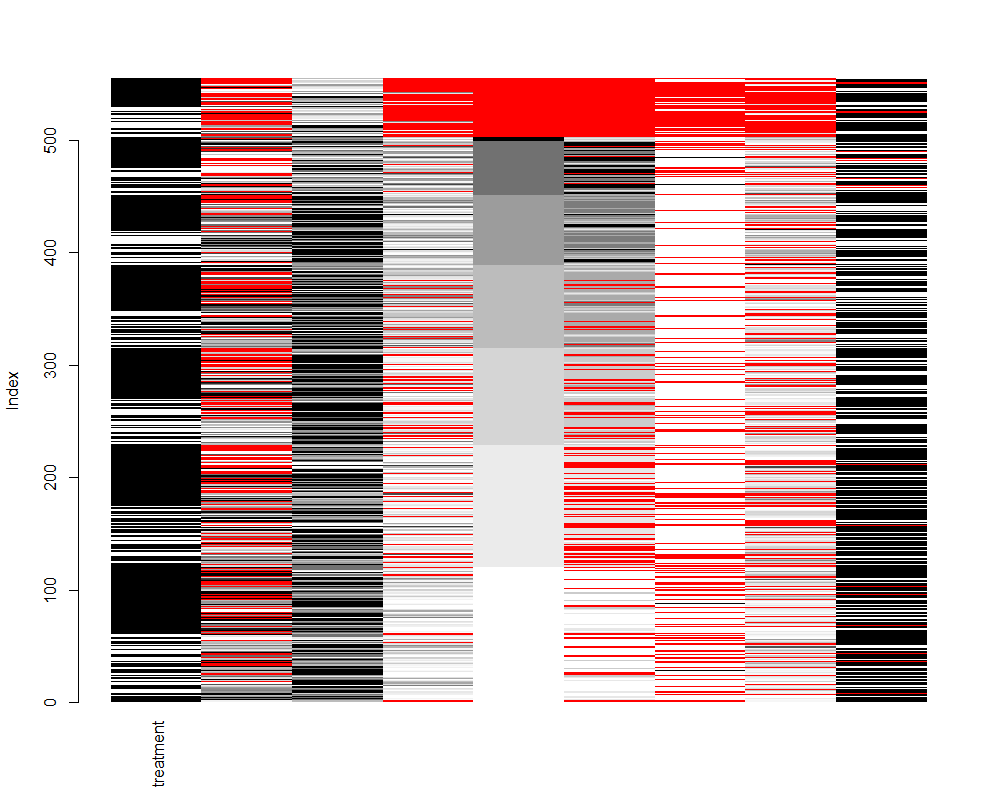  （d） |
| 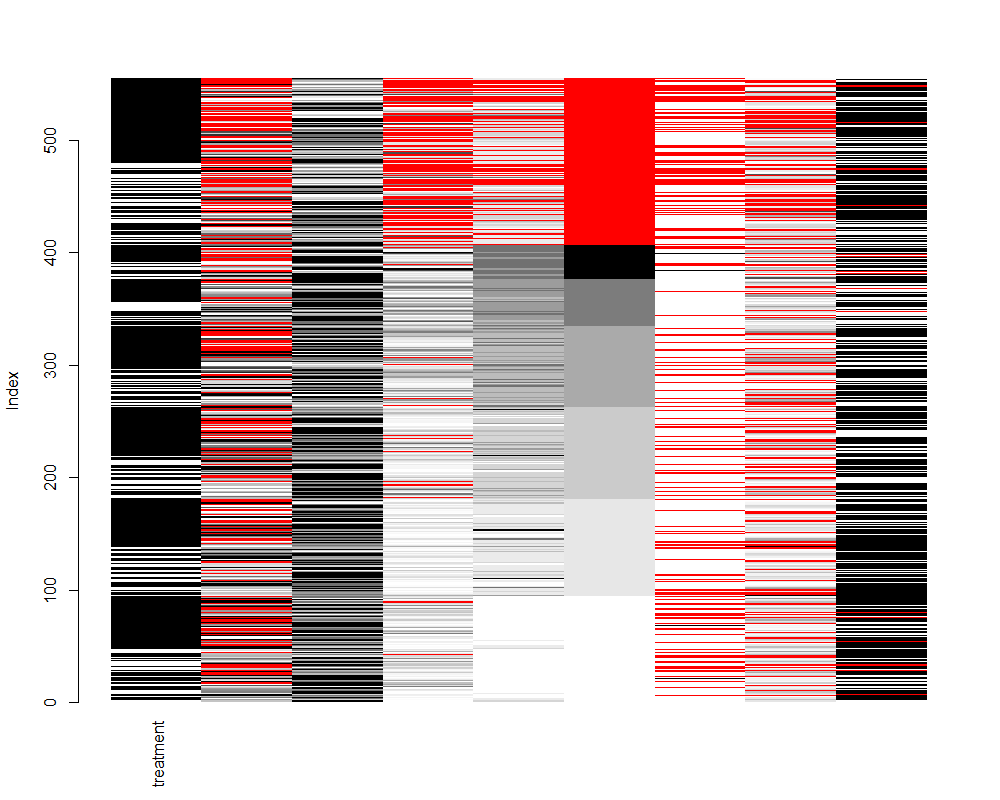  （e） | 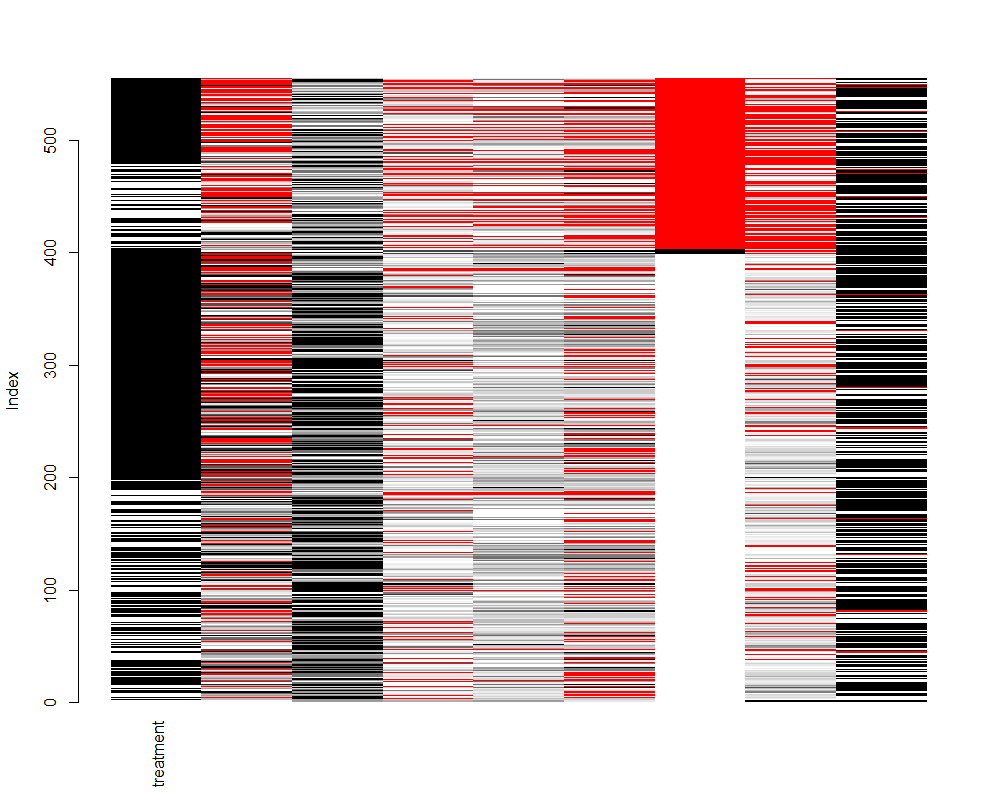  （f） |
| 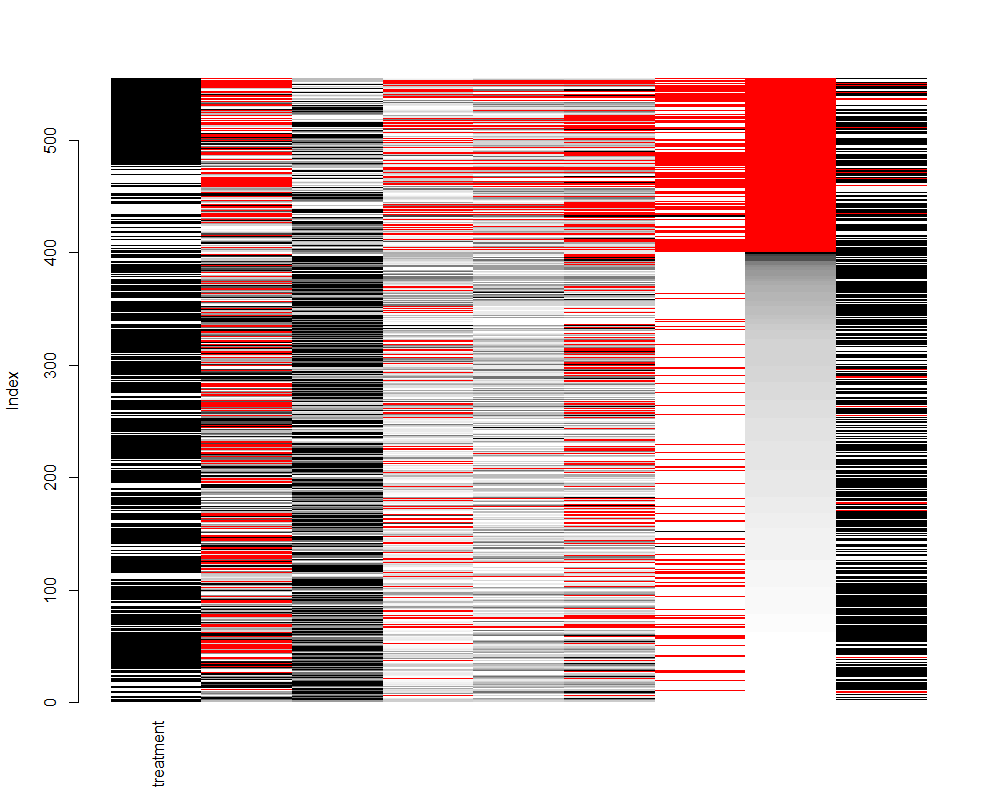  （g） | 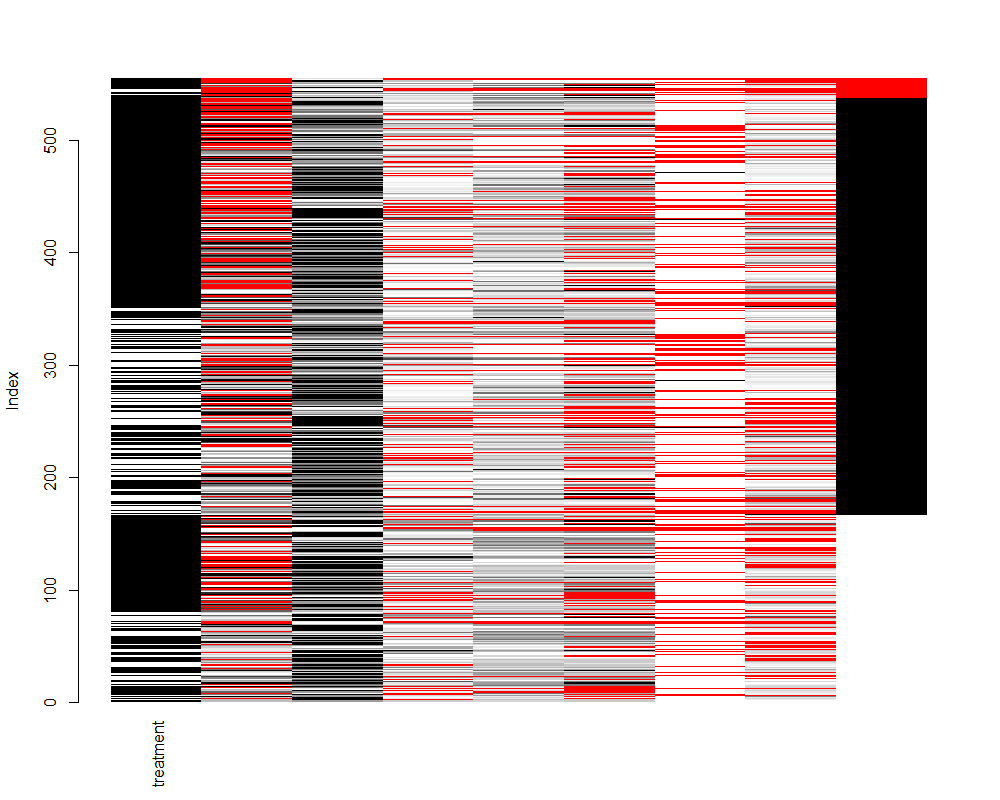  （h） |

Figure 1. Missing data comparison for “de facto” SWD data ^A^

Note：A. Sorted in order of (a)control vs. intervention condition; pre-specified baseline predictors: (b)baseline pharmacy record ; (c) baseline functioning; (d)baseline the overall severity of illness; (e)baseline negative symptoms; (f)baseline substance use; (g)baseline medication side effects, and (h)baseline family supervision. Red indicating missing; gray and black indicating available data; darker color indicating greater value.


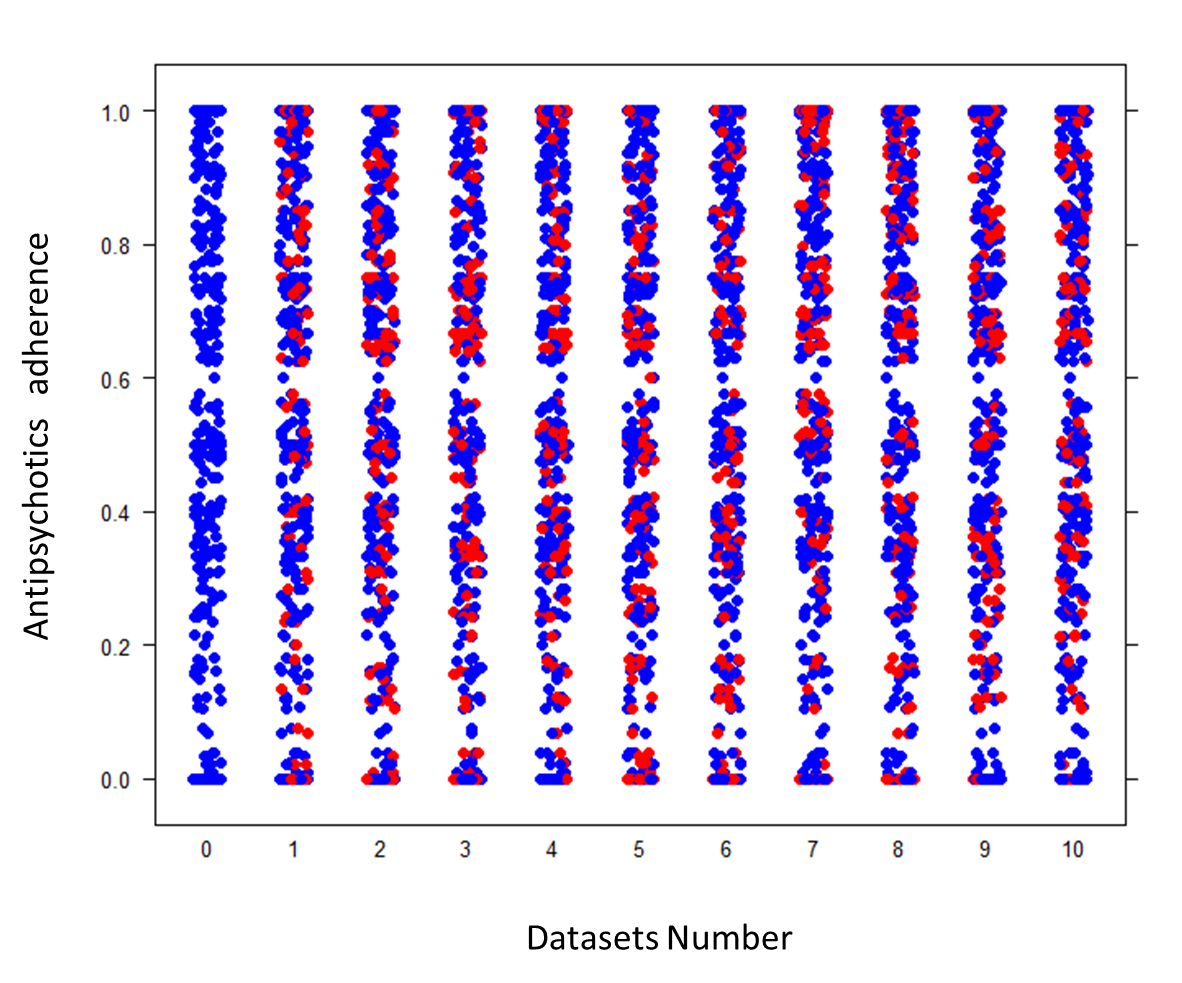


Figure 2. Imputed datasets ^A^

Note：A.: Using medication adherence as an example; 0 represents the original observed data; 1-10 represent datasets after multiple imputations, with the blue dots indicating the original observed values, the red dots representing the imputed values
