# Supplementary material for: Mobile Texting and Lay Health Supporters to Improve Schizophrenia Care in a Resource-Poor Community in Rural China (LEAN Trial): Randomized Controlled Trial Extended Implementation
Source: J Med Internet Res. 2020 Dec 1;22(12):e22631. doi: 10.2196/22631 (PMC7738261; doi:10.2196/22631)
Supplement: Multimedia Appendix 2 [file jmir_v22i12e22631_app2.docx]

# Web appendix

## Appendix 2. Frequency of participants with different Pill-count Adherence in Phase 1 and 3

Further analysis of adherence suggested that the numbers of the participants with perfect adherence (those never missing an antipsychotic dosage) was 40 (17 in the wait-list group, 23 in the intervention group) at the end of Phase 1, and 39 in Phase 3. The numbers of zero adherence (those never taking antipsychotics) increased from 26 (14 in the wait-list group, 12 in the intervention group) in Phase 1 to 41 (all under intervention) in Phase 3. The figure showed the frequency of pill-count adherence in each phase.

| 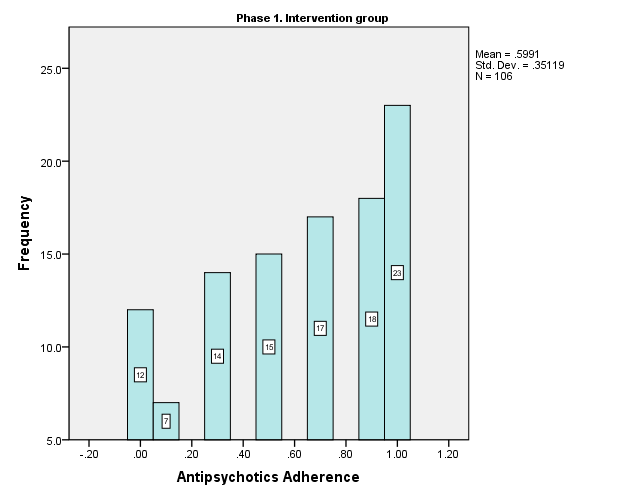  (a) | 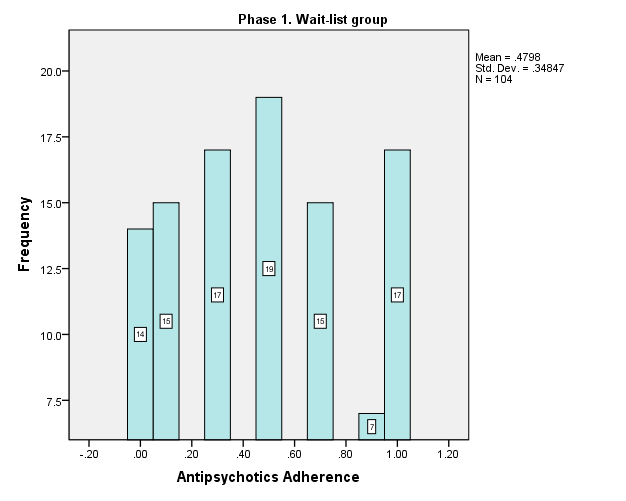  (b) |
| --- | --- |
| 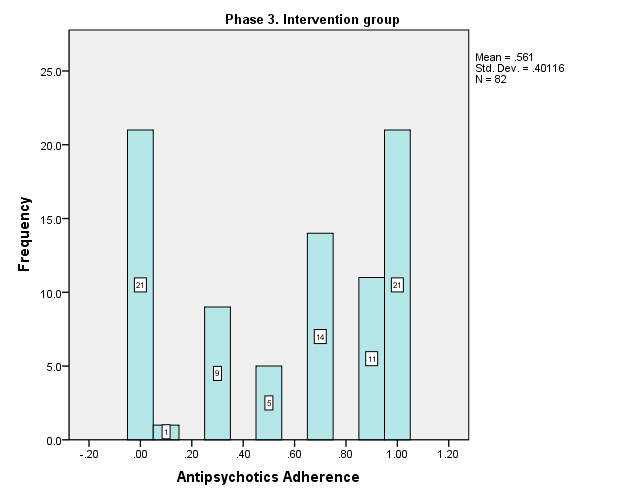  (c) | 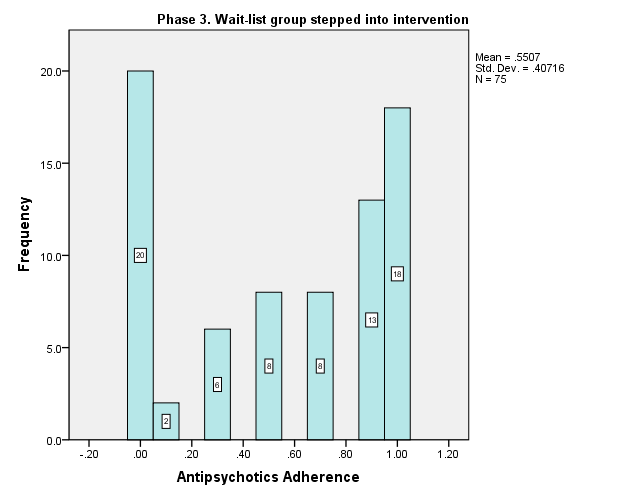  (d) |

Figure. Frequency of participants with different Pill-count Adherence in Phase 1 and 3
