# Supplementary material for: Mobile Texting and Lay Health Supporters to Improve Schizophrenia Care in a Resource-Poor Community in Rural China (LEAN Trial): Randomized Controlled Trial Extended Implementation
Source: J Med Internet Res. 2020 Dec 1;22(12):e22631. doi: 10.2196/22631 (PMC7738261; doi:10.2196/22631)
Supplement: Multimedia Appendix 3 [file jmir_v22i12e22631_app3.docx]

# Web appendix

## Appendix 3. The Discontinuance Reasons for each phase, n(%).

| Discontinuance Reasons | Phase 1 | Phase 3 |
| --- | --- | --- |
|  | N'=26 | N'=41 |
| Discontinuance without capture reasons | 26(100) | 27(66) |
| Feel alleviated | - | 5(12) |
| Refuse to take antipsychotic | - | 3(7) |
| Refuse to take antipsychotic due to side-effects | - | 1(2) |
| Discontinuance due to pregnancy | - | 1(2) |
| Take antipsychotic only when relapse | - | 2(5) |
| Discontinuance when summer | - | 1(2) |
| Discontinuance due to taking other medicine | - | 1(2) |

N’: Number of participants who were discontinuance.
