# Supplementary material for: Mobile Texting and Lay Health Supporters to Improve Schizophrenia Care in a Resource-Poor Community in Rural China (LEAN Trial): Randomized Controlled Trial Extended Implementation
Source: J Med Internet Res. 2020 Dec 1;22(12):e22631. doi: 10.2196/22631 (PMC7738261; doi:10.2196/22631)
Supplement: Multimedia Appendix 4 [file jmir_v22i12e22631_app4.docx]

# Web appendix

## Appendix 4. Relationship between WHODAS interviewees and patients

We calculated the frequency and proportions of the WHODAS questionnaires completed by family members in phase 1 and phase 3. There were 79(30.2%) and 75(31.8%) WHODAS questionnaires were completed by family members respectively. These family members were mainly spouses, parents, children, siblings, and other relatives of the patients. They lived with them and were familiar with the patients' condition. Therefore, it was likely that they could accurately answer questions related to the patients' functional status.

**Table. Relationship between WHODAS interviewees and patients，n(%)**

| Relationship | Phase 1(N=262) | Phase 3(N=236) |
| --- | --- | --- |
| Spouse | 33(41.8) | 28(37.3) |
| Parents | 30(38.0) | 31(41.3) |
| Offsprings | 7(8.9) | 8(10.7) |
| Brothers and Sisters | 5(6.3) | 6(8.0) |
| Others | 4(5.1) | 2(2.7) |
| Amount | 79(100) | 75(100) |
