# Supplementary material for: Mobile Texting and Lay Health Supporters to Improve Schizophrenia Care in a Resource-Poor Community in Rural China (LEAN Trial): Randomized Controlled Trial Extended Implementation
Source: J Med Internet Res. 2020 Dec 1;22(12):e22631. doi: 10.2196/22631 (PMC7738261; doi:10.2196/22631)
Supplement: Multimedia Appendix 5 [file jmir_v22i12e22631_app5.docx]

# Web appendix

## Appendix 5. Sensitivity Analyses

Table 1.Raw versus Adjusted Analysis with covariates and Data imputation for the whole intervention

| Measures | Raw analysis ^A^ | | | | Per protocol analysis^A#^ | | | | Unadjusted Analysis based on  imputation data ^A^ | | | | Adjusted Analysis based on  imputation data ^A^ | | | | Adjusted Analysis based on  imputation data ^B^ | | | |
| --- | --- | --- | --- | --- | --- | --- | --- | --- | --- | --- | --- | --- | --- | --- | --- | --- | --- | --- | --- | --- |
|  | mean difference | 95%CI | | *P*  value | mean difference | 95%CI | | *P*  value | mean difference | 95%CI | | *P*  value | mean difference | 95%CI | | *P* value | mean difference | 95%CI | | *P*  value |
| Pill-count adherence | 0.10 | 0.02 | 0.18 | .01 | 0.10 | 0.02 | 0.18 | .02 | 0.12^C^ | 0.04 | 0.19 | ＜.01 | 0.11^C^ | 0.04 | 0.19 | ＜.01 | 0.11^C^* | 0.02 | 0.20 | .02 |
| WHODAS | 0.02 | -0.02 | 0.06 | .31 | -0.23 | -0.66 | 0.20 | .30 | 0.02^D^ | -0.01 | 0.06 | .23 | 0.02^D^ | -0.01 | 0.06 | .19 | -0.03^D^* | -0.07 | 0.02 | .21 |
| CGI-severity of illness | -0.29 | -0.53 | -0.04 | .02 | -0.27 | -0.51 | -0.03 | .03 | -0.29^E^ | -0.53 | -0.04 | .02 | -0.26^E^ | -0.50 | -0.02 | .04 | 0.07^E^* | -0.22 | 0.35 | .64 |
| CGI-degree of change | 0.09 | -0.11 | 0.30 | .38 | 0.08 | -0.13 | 0.28 | .48 | 0.11^F^ | -0.11 | 0.32 | .32 | 0.10^F^ | 0.00 | 0.25 | .05 | 0.07^F^* | -0.19 | 0.32 | .62 |

Note: A. Analysis based on Phase 1 and Phase 3 data.

1. Analysis based on Phase 1 through Phase 3 data.
2. Adjusted for baseline adherence (pharmacy record), the symptoms, as well as negative symptoms, functioning, substance use, medication side effects, and family supervision.
3. Adjusted for baseline functioning.
4. Adjusted for baseline severity of illness.
5. Adjusted for baseline degree of change.

^#^. Pairs of participants who did not receive texting messages were excluded.

*. Adjusted for the phases and blank phase.

Table 2. Raw versus Adjusted Analysis with covariates and Data imputation on Adherence by different cutoff point for Phase 3

| Measures | Raw analysis | | | |  | Adjusted Analysis with imputation data ^A^ | | | |
| --- | --- | --- | --- | --- | --- | --- | --- | --- | --- |
|  | Relative Risk | 95%CI | | *P* value |  | Relative Risk ^B^ | 95%CI | | *P* value |
| Pill-count adherence |  |  |  |  |  |  |  |  |  |
| cutoff at 0.7 | 2.09 | 1.30 | 3.37 | ＜.01 |  | 1.19 | 1.08 | 1.32 | ＜.01 |
| cutoff at 0.8 | 2.13 | 1.28 | 3.54 | ＜.01 |  | 1.17 | 1.05 | 1.29 | ＜.01 |
| cutoff at 0.9 | 2.25 | 1.29 | 3.94 | ＜.01 |  | 1.16 | 1.05 | 1.27 | ＜.01 |

Note:

A. Step-wedge model-based analysis based on Phase 1 and Phase 3 data.

B. Adjusted for baseline covariates: adherence (pharmacy record), the symptoms, as well as negative symptoms, functioning, substance use, medication side effects, and family supervision.
