# Supplementary material for: Mobile Texting and Lay Health Supporters to Improve Schizophrenia Care in a Resource-Poor Community in Rural China (LEAN Trial): Randomized Controlled Trial Extended Implementation
Source: J Med Internet Res. 2020 Dec 1;22(12):e22631. doi: 10.2196/22631 (PMC7738261; doi:10.2196/22631)
Supplement: Multimedia Appendix 6 [file jmir_v22i12e22631_app6.docx]

# Web appendix

## Appendix 6. Process indicators

In Phase 1, the process indicators were captured from the intervention group only, while in Phase 3, these indicators were captured in all participants. The elements of LEAN in Phase 3 was nearly identical to that in Phase 1^[4, 19]^ except for three factors. First, in Phase 3, 34.1% (92/270) of patient participants and 17.8% (48/270) of lay health supporters without a cellphone did not receive a free phone as the intervention arm did in Phase 1, although only 5.2% (14/270) pairs of patient participants and their lay health supporters had no cellphones. A new carrier policy requesting the in-person authentication for new phone numbers created significant barriers to owning a phone. Second, because of the complete self-withdrawal of antipsychotic medications, or hospitalization due to schizophrenia, some patient participants were asked to receive educational messages only (Table 1). Finally, after Phase 1, upon the request of several participants and/or their health supporters we reduced the frequency of educational messages from daily to every two days. Near the end of Phase 1 and 3, we assessed user experience with LEAN respectively.

In Phase 3, we sent 202 educational text messages and 244 unique SMS reminders. The educational text messages covered self-care, medications, symptoms, relapse prevention, rehabilitation, and social resources. There were a total of 439 educational text messages and 394 reminders in the two intervention periods. Furthermore, during Phase 3, 8 out of 256 (3.13%) participants ceased to receive messages during the program implementation, amongst whom two died and the others felt tired of receiving messages.

According to our texting system log, 74.0% (205/277) of participant pairs in the intervention group in Phase 1, and 65.0% (169/256) of all participants in Phase 3 responded to and confirmed the receipt of the medication reminders by texting back “1”. 26.6%（37/139）pairs responded every day in Phase 1. However, no pairs responded every day in Phase 3. There was 46.8% (65/139) of pairs in Phase 1, and 23.05% (59/256) in Phase 3 who responded at least once over a week. Meanwhile, 4 (4.08%) patient participants and 14 (11.2%) lay health supporters reported that texting bothered them.

During Phase 3, we managed to obtain survey results from 98 (44.4%) patient participants and 125 (56.3%) lay health supporters. Overall the satisfaction of the patients declined substantially from 98.4% (62/63) in Phase 1 to 67.3% (66/98) in Phase 3. Both the patients and the lay health supporters found the texted reminders much more useful than other types of messages. Table 2 presented the results of Phase 1 and Phase 3 survey respectively.

The total cost for a two-period intervention was RMB 118,010 (US$ 17,895), which included RMB 53,000 (US$ 7,433) for texting fees, RMB 11,430 (US$ 1,715) for all customized messages development, RMB 7,200 (US$ 1080) for the message management, and RMB 92,680 (US$ 4,976) for the additional time cost for the health workers. The per capita cost per year was RMB 426 (US$ 61).

The prescribed antipsychotics remained the same throughout the trial (Table 3). The long-acting injectable antipsychotics were not available in the 686 Program at the beginning of LEAN. However, 8 participants received it (haloperidol decanoate) during Phase 3.

Table 1. Message Types received by Participants in Phase 3.,n(%)

| Received Message Type in Participants* | People with schizophrenia | Lay health Supporters |
| --- | --- | --- |
|  | N=270 | N=270 |
| Education messages only | 24(8.9) | 9(3.3) |
| Both Education messages and reminders | 54(57.0) | 213(78.9) |
| None | 92(34.1) | 48(17.8) |
| Note:  N: Number of participants in the cohort who were followed-up.  *: Sample text messages:  **Daily medication reminder:** Good evening! Temple fair in Baisheng town tomorrow and good weather. Please take your medicine and text back “1”.  **Education message (to the lay health supporters):** Note any lack of interest in things that they used to like as it may be an early sign of relapse. We are with you, caring for them.  **Education message (to people with schizophrenia):** Adhering to your medication on time and the prescribed dose is the key to control your symptoms. We are here to help you.  **Education message (to the lay health supporters):** People with schizophrenia may hear voices not heard by others, or think others can see their thoughts, control their thinking, or attempt to harm themselves. Those can lead to fear, withdrawal, or emotional agitation. Try to understand them and get their trust.  **Monitoring messages:** Text 1 if any of the following happens or worsens: problem with sleep, appetite, or concentration; depression; restlessness; tension or nervousness; hearing voices or seeing things that others can’t hear or see; less pleasure gained from things you enjoy; feeling people were watching you; preferring being alone; arguments with others; inability to get your mind off of something. | | |

**Table 2. User experience during Phase 1 and 3,n(%)**

|  | **Patients under intervention** | **Lay health supporters under intervention** |  | **Patients under intervention** | **Lay health supporters under intervention** |
| --- | --- | --- | --- | --- | --- |
| **Phone status** |  |  |  |  |  |
| Used a smartphone | 33(28.9) | 35(33.3) |  | 58(59.2) | 85(68.0) |
| Changed phone number in past 5 months | 13(12.4) | 92(92.0) |  | 11(11.2) | 20(16.0) |
| Phones fully function in past 5 months | 77(77.8) | 92(92.0) |  | 89(90.8) | 120(96.0) |
| **User evaluation** |  |  |  |  |  |
| Overall satisfied with the program | 62(98.4) | 77(100.0) |  | 66(67.3) | 100(80.0) |
| Willing to continue receiving messages | 52(91.2) | 80(94.1) |  | 65(66.3) | 98(78.4) |
| Messages are useful | 61(59.2) | 47(60.3) |  | 58(85.3) | 95(90.5) |
| messages bothered you | 4(6.3) | 9(10.7) |  | 4(4.1) | 14(11.2) |
| Time of texting appropriate | 57(91.9) | 70(90.9) |  | 63(64.3) | 100(80.0) |
| Frequency of texting appropriate | 53(86.9) | 66(83.5) |  | 59(88.1) | 90(84.1) |
| length of messages appropriate | 59(98.3) | 71(92.2) |  | 62(92.5) | 95(91.3) |
| **The most useful part of the messages** |  |  |  |  |  |
| Treatment and medication education | 10(16.9) | 18(24.7) |  | 15(25.0) | 9(9.5) |
| Family care in schizophrenia | 5(5.8) | 8(11.0) |  | 2(3.3) | 2(2.1) |
| Medication reminders | 27(45.8) | 39(53.4) |  | 38(63.3) | 74(77.9) |
| Local news | 2(3.4) | 1(1.4) |  | 2(3.3) | 1(1.1) |
| Weather forecast | 15(25.4) | 7(9.6) |  | 3(5.0) | 9(9.5) |
| **User capability assessment** |  |  |  |  |  |
| Able to navigate phones to read messages | 52(71.2) | 74(84.1) |  | 71(72.4) | 107(85.6) |
| Able to reply messages | 38(52.1) | 55(64.0) |  | 43(43.9) | 63(50.4) |
| Did not understand messages | 12(17.6) | 44(48.9) |  | 9(12.3) | 5(4.7) |
| Some physical disability that prevents using a phone | 12(18.5) | 9(10.7) |  | 0(0.0) | 3(2.4) |
| **User experiences assessment** |  |  |  |  |  |
| Always received messages last month | 44(62.0) | 65(77.4) |  | 72(73.5) | 110(88.0) |
| Always or often read messages | 39(54.9) | 65(76.5) |  | 46(60.5) | 72(64.9) |
| Frequently replied texted reminders | 15(22.4) | 27(31.7) |  | 17(22.4) | 14(13.0) |
| Were concerned about the cost of messages | 7(10.9) | 4(4.8) |  | 1(1.0) | 0(0.0) |

Note: N: Numbers of participants in the cohort with follow-up. N': Numbers of participants who replaying the questionnaire.

Table 3. Prescribed Antipsychotics in Phase 1 and Phase 3

| **Group** | **Prescribe ^A^** | **Phase 1** | **Phase 3** |
| --- | --- | --- | --- |
| **Wait-list group****,n(%)** | |  |  |
|  | Clozapine | 45(33.8) | 42(35.3) |
|  | Risperidone | 43(32.3) | 37(31.1) |
|  | Quetiapine | 25(18.8) | 15(12.6) |
|  | Sulpiride | 25(18.8) | 15(12.6) |
|  | Perphenazine | 15(11.3) | 9(7.6) |
|  | Ripiprazole | 12(9.0) | 10(8.4) |
|  | Olanzapine | 7(5.3) | 0(0.0) |
|  | Chlorpronmazine | 4(3.0) | 1(0.8) |
|  | Perphenazine [Injection] | 2(1.5) | 0(0.0) |
|  | Haloperidol | 1(0.8) | 1(0.8) |
|  | Penfluridol | 1(0.8) | 0(0.0) |
|  | Haloperidol Decanoate [Injection] | 0(0.0) | 4(3.4) |
| **Intervention group,n(%)** | |  |  |
|  | clozapine | 48(35.3) | 41(34.2) |
|  | risperidone | 46(33.8) | 41(34.2) |
|  | quetiapine | 26(19.1) | 18(15.0) |
|  | sulpiride | 21(15.4) | 13(10.8) |
|  | perphenazine | 12(8.8) | 8(6.7) |
|  | aripiprazole | 9(6.6) | 5(4.2) |
|  | Perphenazine [Injection] | 7(5.1) | 0(0.0) |
|  | Olanzapine |  |  |
|  | Ripiprazole | 5(3.7) | 0(0.0) |
|  | Penfluridol | 5(3.7) | 5(4.2) |
|  | Haloperidol | 3(2.2) | 1(0.8) |
|  | Trifluoperazine | 2(1.5) | 0(0.0) |
|  | Haloperidol Decanoate [Injection] | 1(0.7) | 0(0.0) |

Note: A. Those outcomes were tracked by the 686 Program administrative system on a routine basis.
