# Supplementary material for: Mobile Texting and Lay Health Supporters to Improve Schizophrenia Care in a Resource-Poor Community in Rural China (LEAN Trial): Randomized Controlled Trial Extended Implementation
Source: J Med Internet Res. 2020 Dec 1;22(12):e22631. doi: 10.2196/22631 (PMC7738261; doi:10.2196/22631)
Supplement: Multimedia Appendix 7 [file jmir_v22i12e22631_app7.docx]

# Web appendix

## Appendix 7. Cohen’s d effect size of the primary outcome in other research

| **measures** | **Intervention group** | | **Control group** | | **Effect size** |
| --- | --- | --- | --- | --- | --- |
|  | **Mean(SD)** | **n** | **Mean(SD)** | **n** |  |
| Morisky Green Adherence Questionnaire (MAQ) | 1.20(0.10) | 100 | 1.50(0.08) | 154 | 3.24 |
| Psychiatric Medication Adherence Scores (Percent of Doses Taken) ^A^ | 87.5(13.0) | 8 | 83.9(18) | 10 | 0.22 |

Note: A. “TIPS+Text” was regarded as intervention, “TIPS Only” was regarded as control.
